# Pediatric massage therapy for restoring pediatric lung function from COVID-19: A protocol for systematic review and meta-analysis

**DOI:** 10.1097/MD.0000000000021581

**Published:** 2020-08-14

**Authors:** Ke-Lin Zhou, Shuo Dong, Kang Wang, Guo-Bing Fu, Yan Niu, Xiao-Na Xue, Sheng Guo

**Affiliations:** a Dongfang Hospital of Beijing University of Chinese Medicine; b School of Traditional Chinese Medicine, Beijing University of Chinese Medicine; c The Third Affiliated Hospital of Beijing University of Chinese Medicine, Beijing, China.

**Keywords:** COVID-19, complementary and alternative medicine, pulmonary function, pediatric massage, protocol

## Abstract

**Background:**

The novel coronavirus disease 2019 (COVID-19) has caused an international outbreak of a respiratory illness and grown to be a global public health emergency since patients were first detected in Wuhan, China. Given the rapidly growing pandemic and the overwhelmed medical system, there is an urgent need of alternative medicine to help children relieve symptoms during self-quarantine, and possibly to help increase their chances of survival and recovery from COVID-19. By using various manual techniques at specified locations on the surface of the body, pediatric massage manipulation can unblock meridians, promote the circulation of qi and blood and strengthen resistance to pathogens.

**Methods:**

We will search the following electronic databases: Wanfang and Pubmed Database, CNKI, CENTRAL, CINAHL, EMBASE and MEDLINE. Each database will be searched from inception to June 2020. The entire process will include study selection, data extraction, risk of bias assessment and meta-analyses.

**Results:**

This systematic review will evaluate the existing evidence of pediatric massage therapy for restoring pediatric lung function from COVID-19. The outcomes will include the improvement of pulmonary function and adverse effect.

**Conclusion:**

This proposed systematic review will evaluate the existing evidence and explore the potential role of pediatric massage therapy on the effectiveness and safety in pulmonary function of COVID-19 convalescent children.

**PROSPERO registration number:**

CRD42020193396

## 1 Introduction

The novel coronavirus disease 2019 (COVID-19) has caused an international outbreak of a respiratory illness^[1,2]^ and grown to be a global public health emergency since patients were first detected in Wuhan, China.^[3,4]^ Accumulating evidence revealed that COVID-19 causes a broad spectrum of diseases that affects multiple organs including the lung, heart and kidney with reported cardiomyopathy and kidney injury.^[5,6]^ The COVID-19-related disease can lead to pneumonia, acute respiratory distress syndrome (ARDS) and congestive heart failure.^[7]^ Until now, there is no available specific drugs or vaccines can completely cure the patients with COVID-19 infection.^[8]^ Given the rapidly growing pandemic and the overwhelmed medical system, the number of self-quarantined and recovering patients is increasing.^[9]^

Complementary and alternative medicine (CAM) is considered as an adjunct to treat chronic or serious diseases and to self-manage long-term health complaints.^[10]^ Traditional Chinese medicine (TCM), a main form of complementary and alternative medicine, is an ancient and holistic approach to health and healing.^[11]^ Evidence clearly indicated that TCM combined with western medicine can significantly alleviate symptoms of lung function, including decreasing body temperature, cough and breathing difficulties, improving absorption of pulmonary infiltration, and quality of life.^[12]^

Pediatric massage, a form of TCM therapy, aims to manually stimulate specific acupoints of the body which are located primarily on the fingers, palms, arms, head, abdomen and back.^[13]^ Pediatric massage manipulation can unblock meridians, promote the circulation of qi and blood and strengthen resistance to pathogens by using various manual techniques at specified locations on the surface of the body.^[14]^

This systematic review and meta-analysis will summarize the current evidence of pediatric massage in attenuating lung function in children recovering from COVID-19.

## 2 Materials and methods

This systematic review protocol has been registered on PROSPERO (ID: CRD42020193396). The protocol follows the Cochrane handbook for systematic reviews of interventions and the preferred reporting items for systematic reviews and meta-analysis protocol (PRISMA-P) statement guidelines.^[15]^ We will describe the changes in our full review if needed.

## 3 Inclusion criteria for study selection

### 3.1 Type of studies

This review will include clinical RCTs of pediatric massage therapy for lung function in children recovering from COVID-19 without any language or publication status restrictions. Non-RCTs, quasi-RCTs, case series, case reports, crossover studies, uncontrolled trials, and laboratory studies will not be included.

### 3.2 Type of participants

Children diagnosed with COVID-19 of all racial groups and have recovered will be included.

### 3.3 Type of interventions

Interventions will include any type of clinically performed pediatric massage for improvement of pulmonary function in children recovering from COVID-19. This will include Chinese Massage, Japanese Massage, Thai Massage, Swedish Massage, Tuina, Shiatsu, Remedial Massage, General Massage, Acupressure, Reflexology, Manual Lymphatic Drainage. Studies combined with other interventions such as acupuncture, herbal medicines, qigong and yoga will be considered for exclusion.

Control: Treatments other than massage (e.g., usual or standard care, placebo, wait-list controls)

### 3.4 Type of outcome measures

#### 3.4.1 Main outcome(s)

Primary outcomes: The influence of pediatric massage on the pulmonary function and quality of life in convalescent children. Comparison of improvement in main symptoms such as cough and chest tightness before and after treatment, changes in lung imaging, changes in serum leukocyte content; compare the differences in the scores of the World Health Organization's quality of life rating scale (WHOQOL-100) before and after treatment.

Secondary outcomes: Accompanying symptoms (such as myalgia, expectoration, stuffiness, runny nose, pharyngalgia, anhelation, chest distress, dyspnea, crackles, headache, nausea, vomiting, anorexia, diarrhea) disappear rate, negative COVID-19 results rate on 2 consecutive occasions (not on the same day), CT image improvement, average hospitalization time, occurrence rate of common type to severe form, clinical cure rate, and mortality.

#### 3.4.2 Additional outcome(s)

Safety measurements and adverse events.

## 4 Search methods for the identification of studies

### 4.1 Electronic searches

We will search the following electronic bibliographic databases for relevant trials: CNKI (China National Knowledge Infrastructure Database, from 1979 to present); Wanfang Database (from 1990 to present); PubMed Database (from 2000 to present); CENTRAL (Cochrane Central Register of Controlled Trials, from 2000 to present); CINAHL (Cumulative Index of Nursing and Allied Health Literature, from 1937 to present); EMBASE (Excerpta Medica database, from 1947 to present); Ovid MEDLINE ALL (Ovid Medical Literature Analysis and Retrieval System Online, from 1946 to present). There will be no language restrictions.

### 4.2 Data collection and analysis

#### 4.2.1 Study identification

We will use EndNote X9 software to manage the records of searched electronic databases. The initial selection will involve scanning of the titles and abstracts of the retrieved studies. The full text of relevant studies will then be reviewed for study inclusion, in accordance with the inclusion criteria, by 2 authors (KLZ and SD). Potentially relevant articles will be reviewed independently by 2 authors to determine if they meet the prespecified criteria. Any disagreement between authors will be resolved by consensus with a third author. The study selection procedure will follow and be recorded in the PRISMA flow chart. All the evidence will be assessed by the grading of recommendations assessment, development and evaluation (GRADE).

#### 4.2.2 Data extraction and management

According to the inclusion criteria, a standard data collection form will be made before data extraction. The following data will be extracted by 2 authors (KLZ and SD):

*General information*: Research identification, publication year, the title of the study, first author;

*Study methods*: study design, sample size, randomization method, allocation concealment, blinding, incomplete report or selecting report, other sources of bias;

*Participants*: Inclusion and exclusion criteria;

*Intervention*: motion details, treatment duration, and frequency;

*Control*: Type of control methods, motion details, treatment duration, and frequency;

*Outcomes*: Included outcome measures.

#### 4.2.3 Risk of bias assessment

The risk of bias in included studies will be assessed independently by 2 reviewers (KLZ and SD) using the Cochrane Risk of Bias Tool, with any disagreements resolved by consensus or by discussion with a third reviewer. All judgments will be fully described, and the conclusions will be presented in the Risk of Bias figures and will be incorporated into the interpretation of review findings, by means of sensitivity analysis. The risk of bias of each domain will be graded as adequate, unclear, or inadequate. We intend to use the concealment of allocation grading in investigation of any heterogeneity and in sensitivity analysis. Other aspects of study quality including the extent of blinding (if appropriate), losses to follow up, non-compliance, whether the outcome assessment was standardized, and whether an intention to treat analysis was undertaken, will be presented in the risk of bias table describing the included studies and will provide a context for discussing the reliability of the results.

#### 4.2.4 Data analysis

We will use Stata Software [Computer program] (Version 15.1) to process the meta-analysis. Weighted mean difference (WMD) will be used for continuous variable data, and the combined statistical effects of these two are combined. The X^2^ test will be adopted to analyze whether there is heterogeneity in each of the included research questions. I^2^ > 50% is a criterion for significant judgment. The fixed effect model is adopted if I^2^ ≤ 50%, which is considered to have homogeneity between the studies. The random effect model is adopted if I^2^ > 50%, which is considered to have heterogeneity among the studies. The effect size is expressed as 95% confidence interval (CI), and *P* < .05 is considered to be statistically significant.

*Sensitivity analyses:* Heterogeneity may be due to the presence of 1 or more outlier studies with results that conflict with the rest of the studies. We will perform sensitivity analyses excluding outlier studies. In addition, we plan to perform sensitivity analysis to explore the influence of trial quality on effect estimates. The quality components of methodology include adequacy of generation of allocation sequence, concealment of allocation, and the use of intention-to-treat analysis.

*Meta-regression analyses*: If data permits, we will perform the meta-regression analyses.

#### 4.2.5 Publication bias

If sufficient number of trials (more than 10 trials) are found, we will generate funnel plots (effect size against standard error) to investigate publication bias.

#### 4.2.6 Ethics and dissemination

The results of this review will be disseminated through peer-reviewed publication. Because all of the data used in this systematic review and meta-analysis has been published, this review does not require ethical approval. Furthermore, all data will be analyzed anonymously during the review process.

## Acknowledgment

The authors would like to thank all the researchers in our working group.

## Author Contributions

KLZ, SD contributed on methodology and are the guarantors of the review.

KLZ, SD, and SG contributed on data search, analysis, and statistics.

**Methodology:** Ke-Lin Zhou, Shuo Dong.

**Resources:** Sheng Guo.

**Data curation:** Ke-Lin Zhou, Shuo Dong.

**Funding acquisition:** Sheng Guo.

**Project administration:** Shuo Dong.

**Software:** Ke-Lin Zhou, Kang Wang.

**Supervision:** Guo-Bing Fu, Yan Niu, Xiao-Na Xue.

**Writing – original draft:** Ke-Lin Zhou, Shuo Dong.

**Writing – review & editing:** Yan Niu, Xiao-Na Xue, Sheng Guo.
